# Heterogeneous metallaphotoredox catalysis in a continuous-flow packed-bed reactor

**DOI:** 10.3762/bjoc.18.115

**Published:** 2022-08-29

**Authors:** Wei-Hsin Hsu, Susanne Reischauer, Peter H Seeberger, Bartholomäus Pieber, Dario Cambié

**Affiliations:** 1 Max Planck Institute of Colloids and Interfaces, Biomolecular Systems Department, Am Mühlenberg 1, 14476 Potsdam, Germany; 2 Freie Universität Berlin, Institute for Chemistry and Biochemistry, Arnimallee 22, 14195 Berlin, Germany

**Keywords:** flow chemistry, heterogeneous catalysis, metallaphotoredox catalysis, packed bed, photochemistry

## Abstract

Metallaphotoredox catalysis is a powerful and versatile synthetic platform that enables cross-couplings under mild conditions without the need for noble metals. Its growing adoption in drug discovery has translated into an increased interest in sustainable and scalable reaction conditions. Here, we report a continuous-flow approach to metallaphotoredox catalysis using a heterogeneous catalyst that combines the function of a photo- and a nickel catalyst in a single material. The catalyst is embedded in a packed-bed reactor to combine reaction and (catalyst) separation in one step. The use of a packed bed simplifies the translation of optimized batch reaction conditions to continuous flow, as the only components present in the reaction mixture are the substrate and a base. The metallaphotoredox cross-coupling of sulfinates with aryl halides was used as a model system. The catalyst was shown to be stable, with a very low decrease of the yield (≈1% per day) during a continuous experiment over seven days, and to be effective for C–O arylations when carboxylic acids are used as nucleophile instead of sulfinates.

## Introduction

The amount and impact of visible-light-mediated protocols in organic synthesis have increased dramatically since the late 2000s [1]. The main driving force of this phenomenon is the novel reactivity afforded by visible-light photocatalysts that enable new reaction pathways that were previously difficult or impossible to realize [2]. Technical advancements, such as the rise of light-emitting diodes (LEDs) and new reactor technologies were similarly important incentives to popularize light-mediated organic synthesis [3]. The adoption of flow chemistry ensured short photon path lengths and overcame issues related to scalability and productivity caused by the limited light penetration in large batch reactors (Lambert–Beer law), thereby making photocatalysis a promising option for industrially relevant processes [4–5]. This is underlined by several photochemical and photocatalytic transformations that have been performed on industrial scales in continuous-flow reactors [6–8].

**Figure 1:**
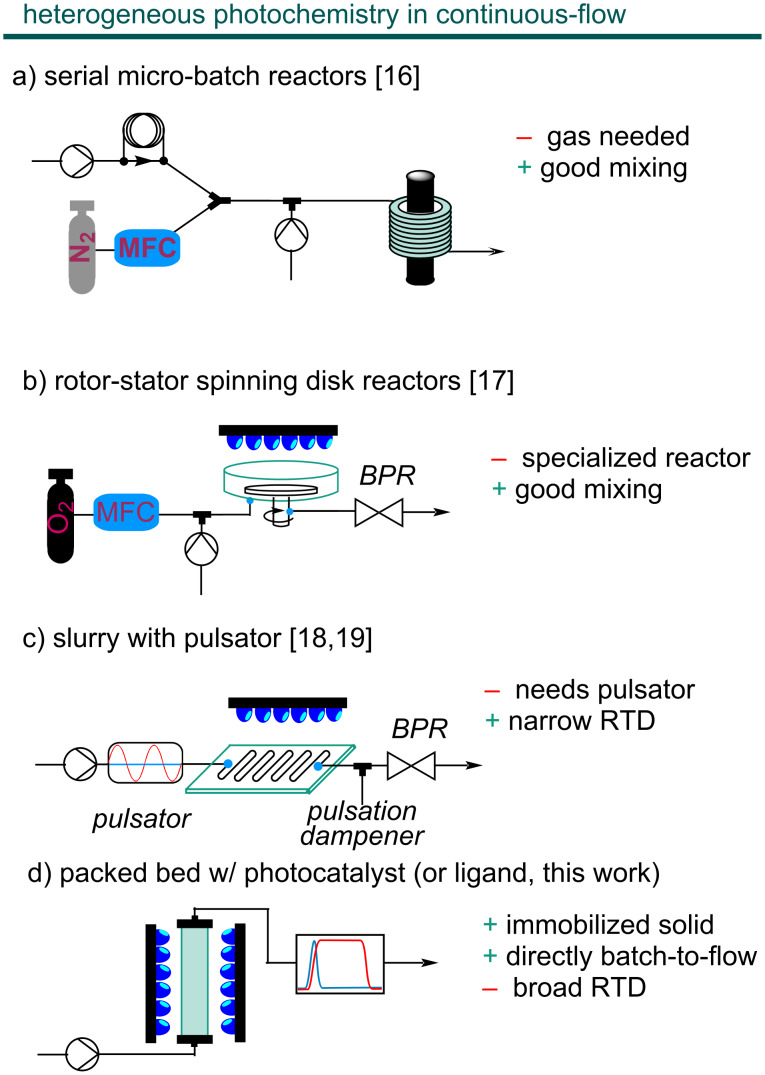
Different approaches to heterogeneous photochemistry in flow. a) Serial micro-batch reactors (SMBR), b) rotor-stator spinning disk reactors, c) slurry with pulsator, d) packed bed.

A particularly appealing branch of photocatalytic organic synthesis is the combination with other modes of catalysis in dual catalytic approaches [9]. Especially the combination with other transition metal catalysts (metallaphotoredox catalysis), such as nickel complexes, resulted in a vast number of new methods to achieve cross-couplings under mild conditions [10]. However, the conditions of these methods are often hard to translate to flow [11–12] and significant changes to the optimized batch protocol are usually required [13–14]. The most common obstacle in the batch-to-flow translation of metallaphotoredox reactions is their frequent heterogeneous nature, most commonly due to poorly soluble inorganic bases, catalysts or additives [5,15]. Solid reagents and catalysts cause severe problems, such as reactor clogging under continuous-flow conditions. To prevent reactor fouling in (gas-)solid-liquid heterogeneous photoreactions, different solutions have been proposed [16], including the use of serial micro-batch reactors (SMBR, Figure 1a) [17], rotor-stator spinning disk reactors (Figure 1b) [18], and the combination of oscillatory pumps with microstructured reactors (Figure 1c) [19–20].

When the (photo)catalyst is the solid material in a heterogeneous reaction, packed-bed reactors are the most appealing solution for flow processes [5] (Figure 1d): The heterogeneous catalyst remains located in a specific part of the reactor through which the reaction mixture is pumped, which reduces material damage through attrition and the confinement of the catalysts in the packed bed lifts the need for solid separation. If the catalyst is sufficiently (photo-)stable, a higher turnover number can be achieved [16]. Issues related to the low surface-to-volume ratio that prevents efficient irradiation of heterogeneous photocatalysts in packed beds can be addressed by adding, for example, glass beads [21]. These considerations have justified the development of several strategies to immobilize transition-metal photocatalysts [22].

**Figure 2:**
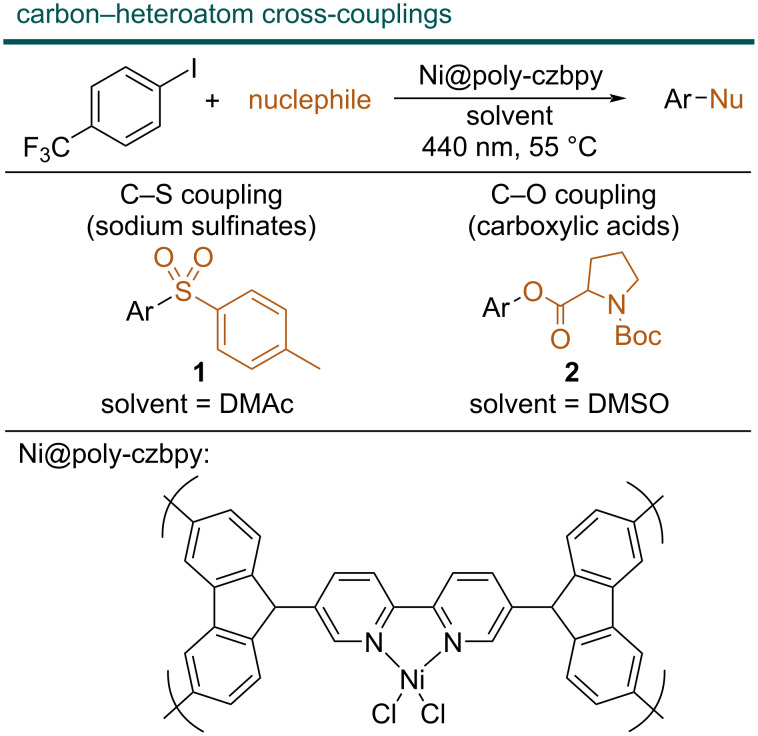
Light-mediated carbon–heteroatom cross-couplings. The yields reported are the NMR yields obtained in flow with the optimized conditions described later. DMAc = dimethylacetamide, DMSO = dimethyl sulfoxide.

In the case of flow-metallaphotoredox catalysis packed-bed reactors were not applied to date. This is likely because these reactions are mainly carried out using homogeneous catalysis. Several studies have shown that the combination of solid photocatalysts (i.e., semiconductors) and homogeneous nickel complexes are feasible, but the fact that the nickel complex is in solution reduces the benefits of packed-bed reactor types [19,23–24]. Recently, several bifunctional heterogeneous catalysts that combine the photo- and the nickel catalyst in a single material have been reported [23,25–27]. For example, some of us have shown that a bipyridine ligand decorated with two carbazole groups can be polymerized to afford a heterogeneous macroligand (poly-czbpy) that coordinates nickel and serves as an active catalyst for light-mediated carbon–heteroatom cross-couplings of sodium sulfinates, carboxylic acids and sulphonamides with aryl halides (Figure 2) [28]. Although recyclable, batch reactions are characterized by long reaction times (24 h).

Here, we present a detailed investigation of a continuous-flow strategy for these heterogeneous catalysts, using a packed-bed reactor. The use of a packed-bed reactor for these solid–liquid reactions is attractive as reaction and separation can be combined in one step. This is particularly notable in our case since, after complexing poly-czbpy with nickel (Ni@poly-czbpy), the simultaneous separation of both the photocatalyst and metal-catalyst is achieved. The combination of both catalytic activities in a single material is crucial to obtain this result, as a mixture of a heterogeneous photocatalyst with an immobilized metal catalyst would be problematic both in terms of packed bed uniformity and activity, while partially homogeneous systems would need downstream separations. We used in-line reaction monitoring to study several process parameters, such as time, temperature and the photon flux, to maximize the throughput and evaluate the long-term stability of this catalytic approach.

## Results and Discussion

### Reactor assembly and model reaction

We started our investigations by preparing a packed-bed reactor using a glass column (6.6 mm i.d., 100 mm length) that can be used in a dedicated setup for heterogeneous flow photocatalysis carried out in a commercial photochemical flow reactor (from Vapourtec) [29–32]. To decrease the optical density of the bed, the column was loaded with a mixture of poly-5,5’-di(9*H*-carbazol-9-yl)-2,2’-bipyridine (poly-czbpy), glass beads and silica [33]. Once the column was ready, nickel was ligated to the polymerized ligand to afford the complexated catalyst (Ni@poly-czbpy). Based on previous batch optimizations we aimed for a ligand/metal ratio of 2:1 to ensure no unligated nickel is present as it negatively impacts the selectivity. By recirculating a solution of NiCl_2_∙glyme (4.3 mM) through the reactor for three hours (flow rate: 0.5 mL/min) most of the Ni was ligated to the macroligand (84% by ICP, see Supporting Information File 1).

**Figure 3:**
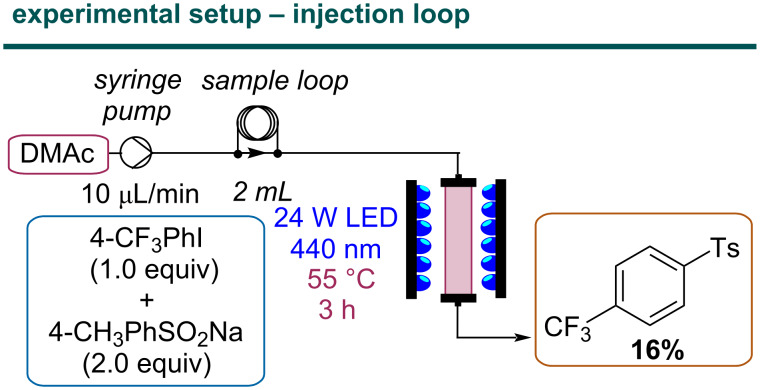
Flow diagram of the experimental setup loaded in an injection loop with the reaction mixture.

To test the activity of the bed, a flow setup consisting of a syringe pump, a sample loop for injecting low volumes of the reaction mixture, and the photoreactor unit was assembled (Figure 3). The C–S coupling between 4-iodobenzotrifluoride and sodium *p*-toluenesulfinate was chosen as the model reaction [28]. In contrast to other protocols, this reaction does not require any additives, such as a base, which allows for a straightforward proof-of-principle study on the long-term stability of the polymeric material under flow conditions. Compared to the original batch procedure, a reduction of the reaction concentration by a factor of two was necessary to ensure complete solvation of the sulfinate salt.

Once homogeneous conditions were achieved, the reaction mixture was injected into the reactor, which was radiated with 440 nm LEDs at 55 °C. A poor yield (16%) was obtained using a residence time of 3 hours. This result called for a more detailed investigation of crucial reaction parameters to understand if the limitation is of catalytic or technological nature.

### Steady-state and automated reaction analysis

**Figure 4:**
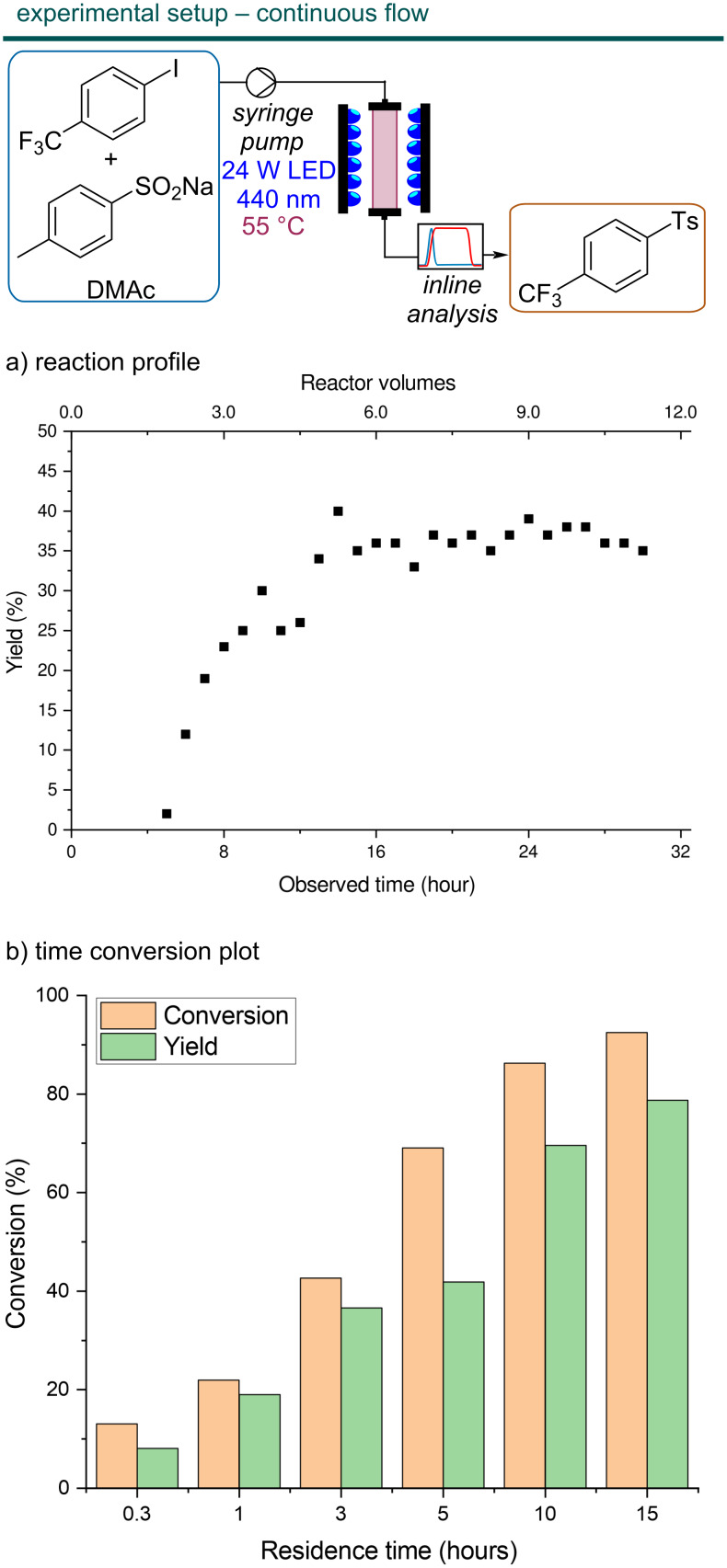
Flow diagram of the experimental setup adopted and time necessary to obtain steady-state conditions. (a) Reaction profile for a residence time of 3 hours. Stable conditions are obtained after about 12 h, because of the low flow rate some delay is also due to dead volume between the reactor and the NMR spectrometer used for reaction monitoring. For example, the NMR flow cell volume, about 1 mL, is alone responsible for 100 minutes of delay. (b) Time conversion plot, almost full conversion is observed after 15 hours, comparable with batch results (22 h).

To systematically study the cross-coupling using the packed-bed reactor, we decided to equip the continuous-flow setup with a dedicated tool for in-line analysis. Such techniques enable rapid investigations of process-related parameters [34]. In particular, the presence of a trifluoromethyl group in the substrate enabled straightforward reaction monitoring via ^19^F NMR. To this end, a 1 T benchtop NMR equipped with a flow cell was connected to the reactor outlet and used to acquire a series of spectra. In particular, a series of 128 repetitions with a 90° pulse width and a relatively long repetition time of 5.2 s (3.2 acquisition + 2s delay) was used to ensure accurate integrals. The processed FIDs were integrated and the following integration limits were used: starting material between −60.85 to −61.1 ppm, product between −61.1 and −61.35 ppm and an unidentified side-product at −60.5 to −60.7 ppm, no other peaks were detected in the fluorine spectra. The product yield calculated from the relative ratio of the product to the total integral area was comparable with the NMR yield calculated with hexafluorobenzene as internal standard in the high-field spectrometer (see Supporting Information File 1, Table S1). With the in-line analytical data in hand, it was clear that the sample loop volume was too low to reach steady-state conditions. By switching to a continuous operation mode, we realized that a residence time of around 3 hours was necessary to reach steady-state conditions and the yield improved significantly (36%) (Figure 4a). This corresponds to approximately five reactor volumes, even though the volume of the NMR flow cell and the tubing between the reactor and the flow cell is also responsible for this delay.

As a next step, we intended to perform a residence time screening. However, the five reactor volumes needed to reach steady-state conditions meant that, depending on the residence time, up to several hours were needed to acquire each data point. To accelerate the acquisition of experimental results, we decided to equip our reactor with an in-line benchtop NMR spectrometer at the reactor outlet. First, we verified that the NMR yield calculated directly from the benchtop ^19^F NMR spectrum was in good agreement with the high-field NMR yields calculated with an internal standard (see Supporting Information File 1, Table S1). Then, a program was developed to monitor the reaction yield over time by automatically acquiring, processing and integrating the ^19^F NMR spectrum of the reaction mixture flowing in the spectrometer (see relevant code in Supporting Information File 2). In particular, the python packages flowchem [35] and nmrglue [36] were used to control the spectrometer and process the free induction decay (FID) files, respectively. The reaction was run until stable conditions were obtained, defined as seven consecutive spectra in which the coefficient of variation (CV) of the auto-integrated product yield was below 3%.

### Residence time, photon flux and temperature studies

Having developed an automated analysis system, we proceeded with a residence time screening. A reaction time of 15 hours was necessary to reach almost quantitative conversion (92%) (Figure 4b). These results suggest that neither a higher local concentration of light-absorbing species nor the improved light distribution significantly improves the transformation compared with the batch reaction.

Next, we investigated if the reaction rate significantly depends on the received photon flux (Table 1). For these studies, we chose a residence time of 3 hours (i.e., 10 µL/min flow rate) as a compromise between conversion (high enough to observe changes with the different conditions tested) and residence time (as short as possible to reduce the amount of time needed for the experiments). Changing the light intensity had a minor impact on the reaction rate (Table 1, entries 2–4). This observation suggests that the turnover determining step is likely not of photochemical nature.

**Table 1:** Optimization of temperature and light intensity for the coupling of 4-iodobenzotrifluoride and sodium *p*-toluenesulfinate.

Entry^a^	Light intensity	Temp. [°C]	Conversion [%]	Yield [%]

1	0%	55	0	n.d.
2	50%	55	66	61
3	75%	55	67	62
**4**	**100%**	**55**	**72**	**67**
5	100%	40	60	54
6^b^	100%	70	64	55
7^c^	100%	55	62	51

^a^Data was collected by 37.6 MHz ^19^F NMR; ^b^the colour of the polymer in the column turned dark during irradiation (see Supporting Information File 1, Figure S4); ^c^the data was collected after entry 5.

Based on this consideration, we turned to the reactor temperature as a means for process intensification. As expected, performing the reaction at a lower temperature proved detrimental (Table 1, entry 5). However, at higher temperatures, the colour of the polymer in the packed bed turned rapidly black (see Supporting Information File 1, Figure S4), and lower yields were observed (Table 1, entries 6 and 7). The catalyst deactivation could be due to the formation of nickel-black [28], or via (photo-)thermal degradation of the polymer.

### Packed-bed stability

**Figure 5:**
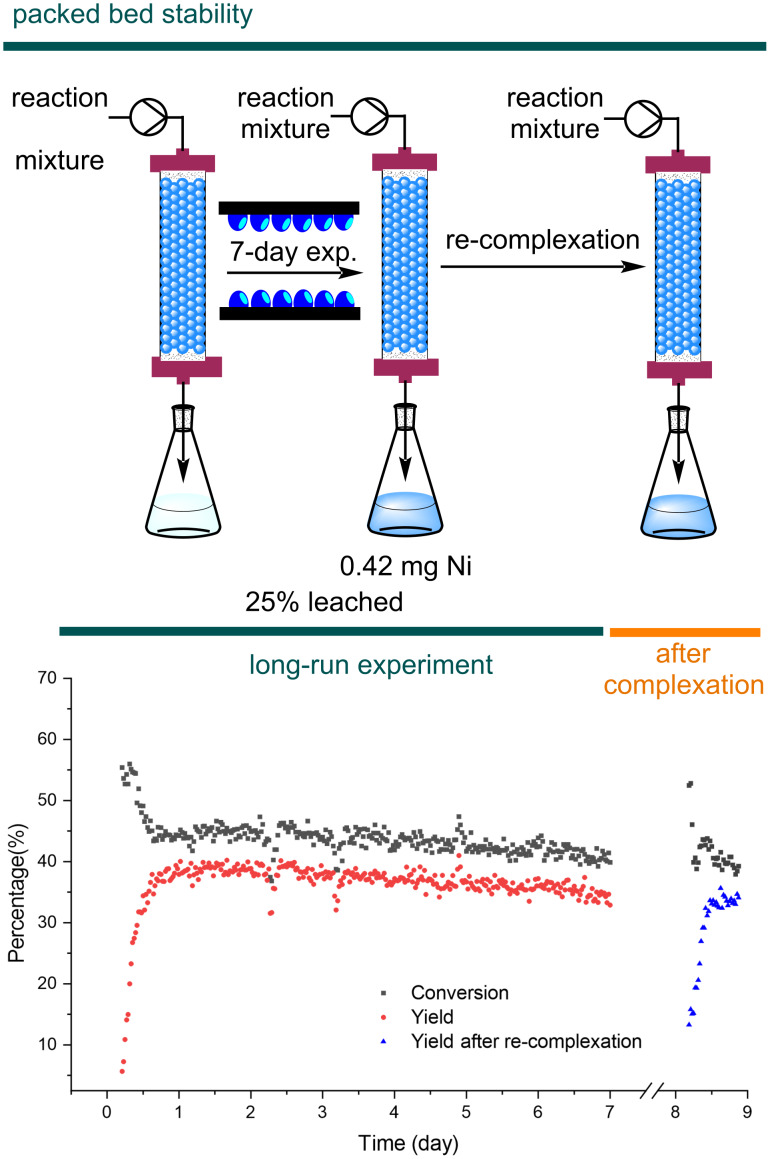
The production campaign of **1** for a seven day experiment.

To study the stability of the Ni@poly-czbpy packed-bed reactor and evaluate its suitability for scaling-out, a continuous experiment over seven days was performed using the conditions with 100% intensity, 55 °C and 3 hours residence time (Figure 5). After reaching steady-state conditions (12 h, in agreement with previous observations, see Figure 4a), only a minor decrease in the catalyst activity (about 1% per day) was observed throughout the experiment, demonstrating the good long-term stability of the heterogeneous catalyst. In particular, the catalyst turnover number (TON) calculated over the 7-day experiment is comparable with the TON observed in batch for a single reaction (35 vs 36, respectively). Since the catalyst is still highly active after 7 days, a higher TON could be achieved by extending the experiment duration. This observation constitutes a promising starting point for applications in large-scale synthesis or automated reaction optimization.

Based on literature precedents on catalyst leaching in packed-bed reactors [37–38], we assumed that the decreased activity could be linked to nickel leaching. Based on ICP results on the reactor outlet collected in the long-run experiment, after seven days of continuous reaction 0.42 mg of nickel (25% of the 1.66 mg initially complexed, see Figure 5) leached into the reaction solution. However, performing another round of complexation with NiCl_2_∙glyme did not restore the original activity of the catalyst in the packed bed. Together with the catalyst deactivation observed in the temperature study, this result points at a temperature-dependent ligand photodegradation as a likely deactivation mechanism. The amount of nickel leaching observed is significant and, depending on the substrate, the metal contamination after chromatography might still be too high for use as API. If the remaining nickel content becomes an issue, a column packed with an immobilized scavenger could be used to further reduce the Ni content in the final product [39].

### Reactor optimization

**Figure 6:**
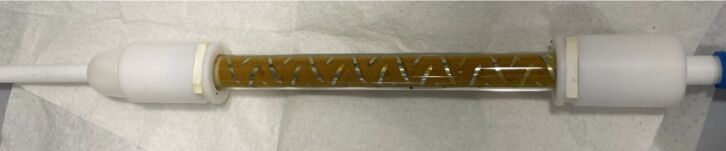
Photo of the packed column with a helical static mixer (polished SS316, 10 cm length, 15 mixing elements L/D = 1.04 from Stamixco AG).

Flow maldistribution and poor mixing efficiency in the packed bed could cause the relatively long time necessary to reach steady-state conditions. Consequently, we evaluated a static mixer to improve the flow distribution in the packed bed [40–42]. The residence time distribution (RTD) of the reactor was measured via a pulsed tracer experiment (see Supporting Information File 3 for details) and compared with a modified reactor unit containing a helical static mixer (Figure 6) [43]. The addition of the static mixer had a limited impact on both the standard deviation of the mean residence time and the reaction outcome, most likely due to the low flow rate (10 μL/min) [43–44]. An alternative approach to obtain a narrower RTD is the reduction of the reactor diameter, as this would decrease the axial dispersion [45]. Replacing the glass column (i.d. 6.6 mm) with a PTFE capillary with a smaller inner diameter (i.d. 5/32”, 3.9 mm) resulted in a narrower residence time distribution and higher yields (see Table 2).

**Table 2:** Comparison of different reactors.

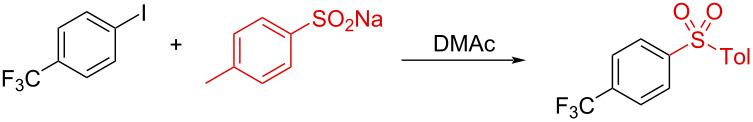				

Entry	Reactor	Conversion [%]	Yield [%]	Mean residence time ± standard deviation [min]

1^a^	glass column	45	36	161 ± 58
2^b^	glass column + static mixer	45	37	171 ± 42
3	5/32” ID tube^c^	57	50	195 ± 32

^a^6.60 mm ID; ^b^outfit of the reactor (Figure 6) ^c^5/32” equals to 4.0 mm.

### C–O coupling reaction

**Scheme 1:**
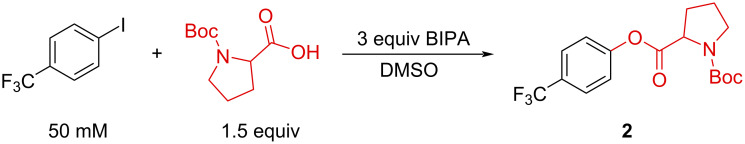
C–O coupling between 4-iodobenzotrifluoride and *N*-(Boc)-proline.

Finally, we evaluated the use of the capillary-based reactor for the related C–O coupling of 4-iodobenzotrifluoride and *N*-(Boc)-proline with *N*-*tert*-butylisopropylamine (BIPA) in dimethyl sulfoxide (DMSO) (Scheme 1). In analogy with the C–S coupling, a residence time of 3 hours was chosen for a test experiment, resulting in 81% conversion and 61% NMR yield. In this case a significant acceleration compared to the original batch reaction time (24 h) was observed, likely thanks to the use of the same reaction concentration as in the original batch report [28]. This was unlike the C–S coupling, where the limited solubility of the sulfinate salt required a dilution of the reaction conditions to obtain a homogenous reaction mixture. As previously observed [28], the reaction concentration has a significant impact on the efficiency of the nickel cycle in metallaphotoredox reactions. It is therefore not surprising that a larger acceleration of the reaction kinetics in flow versus batch was observed for the C–O coupling as opposed to the C–S coupling.

## Conclusion

In summary, we developed a packed-bed reactor for metallaphotoredox catalysis in continuous flow. The heterogeneous catalyst used, based on a bipyridine ligand decorated with two carbazole groups, served as both photo- and nickel catalyst, making the reactor packing simple and reproducible. Compared with homogeneous approaches to metallaphotoredox catalysis, this heterogeneous solution simplifies the catalyst separation and the translation of the optimized batch conditions to flow. Most notably, reactions previously optimized in batch could be performed in continuous flow directly with little (C–S coupling) to no (C–O coupling) changes to the reaction conditions. Overall, the lack of catalyst separation and the possibility of combining the reactor with in-line analytical feedback enables the flow synthesis of C–S and C–O coupled products in a simple, versatile and amenable to automation way.

## Supporting Information

File 1 Details of packed-bed assembly, experimental procedures, reaction optimization and compounds characterization data.

File 2 Residence time distribution calculation notebook.

File 3 NMR control and auto-integration notebook.
