# Supplementary material for: Heterogeneous metallaphotoredox catalysis in a continuous-flow packed-bed reactor
Source: Beilstein J Org Chem. 2022 Aug 29;18:1123–30. doi: 10.3762/bjoc.18.115 (PMC9443413; doi:10.3762/bjoc.18.115)
Supplement: File 1 — Details of packed-bed assembly, experimental procedures, reaction optimization and compounds characterization data. [file Beilstein_J_Org_Chem-18-1123-s001.pdf]

BEILSTEIN JOURNAL OF ORGANIC CHEMISTRY

Supporting Information

for

Heterogeneous metallaphotoredox catalysis in a continuous-flow packed-bed reactor

Wei-Hsin Hsu, Susanne Reischauer, Peter H. Seeberger, Bartholomäus Pieber and Dario Cambié

Beilstein J. Org. Chem. **2022,** *18,* 1123–1130. doi:10.3762/bjoc.18.115

Details of packed-bed assembly, experimental procedures, reaction optimization and compounds characterization data

License and Terms: This is a supporting information file under the terms of the Creative Commons Attribution License (https://creativecommons.org/licenses/by/4.0). Please note that the reuse, redistribution and reproduction in particular requires that the author(s) and source are credited and that individual graphics may be subject to special legal provisions.
The license is subject to the *Beilstein Journal of Organic Chemistry* terms and conditions: (https://www.beilstein-journals.org/bjoc/terms)

Table of Contents

1. General remarks S1
2. Flow setup S2
3. Preparation of the packed bed reactor S3
4. General reaction procedure S4
5. Reaction optimization S6
    - 5.1. Flow setup (injection loop vs. continuous operation) S6
    - 5.2. Conversion vs. time S7
    - 5.3. Optimization of temperature and light intensity S7
    - 5.4. Catalyst loading S8
6. Residence Time Distribution analysis S9
7. Nickel content S9
8. Compound characterization S11
9. NMR spectra S14

1. General remarks

All Chemicals and solvents were acquired from Sigma Aldrich and used without further purification. Analytical thin layer chromatography (TLC) was performed on pre-coated TLC-sheets, ALUGRAM Xtra SIL G/UV254 sheets (Macherey-Nagel) and visualized with 254 nm light. ^{1}H-, ^{13}C-, and ^{19}F- spectra for compound characterization were recorded on a Varian 400 spectrometer (400 MHz, Agilent), the spectrometer used for in line experiment was a Spinsolve (40 MHz, Magritek). Purification of final compound was carried out by flash chromatography using silica gel pore size 60 Å 230–400 mesh particle

size (Sigmal Aldrich #60752). Inductively coupled plasma-optical emission spectroscopy was carried out using an ICP-OES 715 ES (Varian, USA) after digesting the polymers in a mixture of nitric acid and sulfuric acid (v/v = 2:1). The static mixer used was an Helical Static Mixer with 15 mixing elements (L/D = 1.04), polished surfaces, Length = 100 mm in stainless steel 316L (Cat No. HT50-6.4-15-316L) from Stamixco AG (Switzerland). The poly-czbpy polymer was synthesized as described in reference 28 of the manuscript.

2. Flow setup

A commercially available Vapourtec R-series equipped with an immobilized photoreactor module was employed in this study. The photoreactor module is comprised of a 440 nm LED (input power 60 W, radiant power 24 W) that irradiates a cavity that hosts a 100 mm long glass column (Omnifit, 6.6 mm inner diameter i.d.) packed with the polymeric ligand. The reaction mixture was charged in two glass syringes loaded on a syringe pump (Harvard Apparatus Elite11). The outlet of the two syringes, combined with a Tee piece, was routed to the Vapourtec pressure sensor with PTFE tubing (1/16” OD, 1 mm i.d.) to monitor the reactor pressure drop and then to the column inlet. The reactor outlet was optionally connected with a Magritek Spinsolve benchtop NMR for the experiment with in-line analysis and then to a multiposition valve (Knauer V2.1S) controlled via ethernet to collect and fractionate the reactor outlet into different vials.

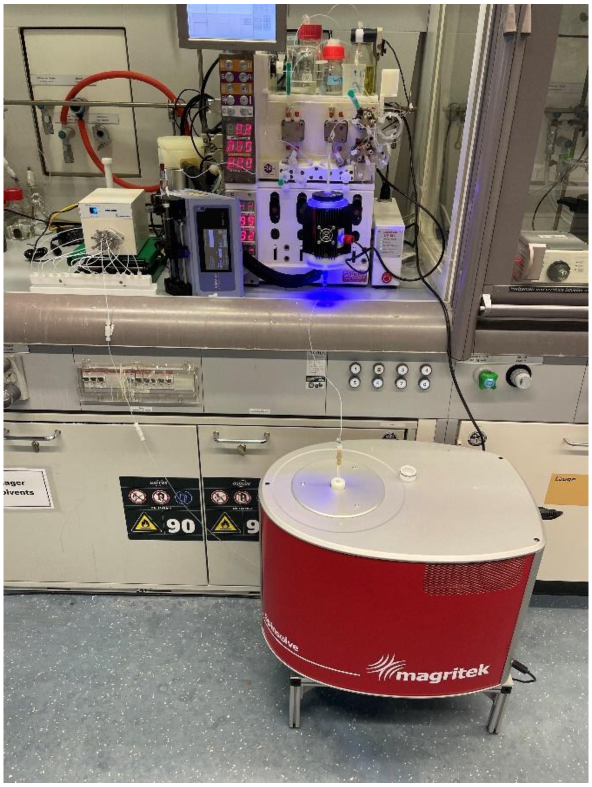

Figure S1: Experimental setup used for the flow experiments. The immobilised photoreactor module (in black) is connected to the bay 3 of the VapourtecR4, the blue halo is the marginal stray light escaping from the light filters at the top and bottom of the column holder. The syringe pump in purple is used to pump the reaction mixture, that is routed to the R4 pressure sensor, photoreactor module before flowing through the flow cell of the NMR and reaching the multi-position valve (in white, on the left) where the reactor outlet is fractionated into different vials for collection.

3. Preparation of the packed bed reactor

A 1.6 wt % poly-czbpy mixture was prepared by mixing in an agate mortar 35 mg of poly-czbpy with 665 mg of silica (230–400 mesh particle size, Sigma-Aldrich Cat no. 60752), starting with a 1:1 ratio and progressively adding more silica until a uniform mixture was obtained. The resulting yellowish powder was further mixed with glass beads (1.4 g, 250 µm acid-washed/silanized, Sigma-Aldrich Cat no. 59203). The glass column was plugged with a small cotton filter, charged with 2.0 g of the glass bead/poly-czbpy/silica mixture and equilibrated with dry dimethylacetamide (DMAc). A solution of 7.5 mg of $NiCl_2$·glyme (about 0.5 equiv. vs. the bipyridine, calculated from the polymer mass based on the monomer molecular weight) in 8 mL of dry DMAc was used to load the nickel in the

column by recirculating it for 3 hours, resulting in the active Ni@poly-czbpy. The glass column reactor dead volume was measured in 2.0 mL.

For glass column with static mixer and 5/32" i.d. tube, same ratio (1.6 wt %) was prepared and same amount of materials (2.0 g) was packed to the reactors (Figure S2).

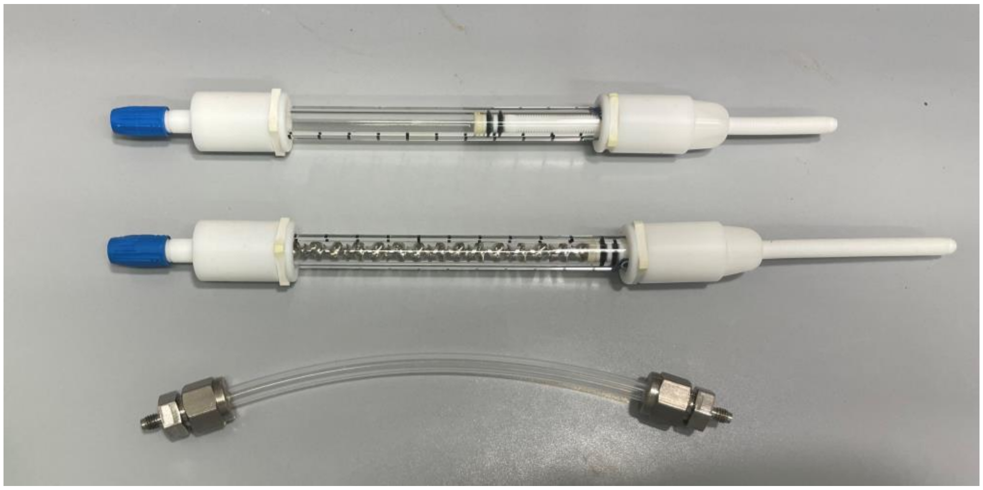

Figure S2: Three reactors without packed material. The difference of the length of column due to the difference of the inner diameter reactor.

4. General reaction procedure

A solution of 4-iodobenzotrifluoride (25 mM) and sodium *p*-toluenesulfinate (50 mM, 2 equiv.) was prepared in *N,N*-dimethylacetamide (DMAc) for the C–S coupling reaction. The reaction mixture was sonicated to accelerate the slow dissolution of the sulfinate until a clear solution was achieved (generally 5 to 10 min). The reactor temperature and light intensity were controlled directly from the Vapourtec R4 reactor, while the flow rate was set on syringe pump (Harvard Apparatus Elite 11) used for the infusion. Unless otherwise specified, a temperature of 55 °C and a 100% light intensity were used. In-line ^{19}F NMR spectra were automatically acquired by a Magritek Spinsolve 40 MHz spectrometer controlled by a custom Python script (SI3, NMRcontrol_autointegration pdf). The integral range of 4-iodobenzotrifluoride as starting material was −60.85 to −61.1, and desired product 1 was −61.1 to −61.35 (Figure S3). The reaction was run until stable conditions were obtained, defined as 7 consecutive spectra in which the coefficient of variation (CV) of the auto-integrated product yield was below 3.0%.

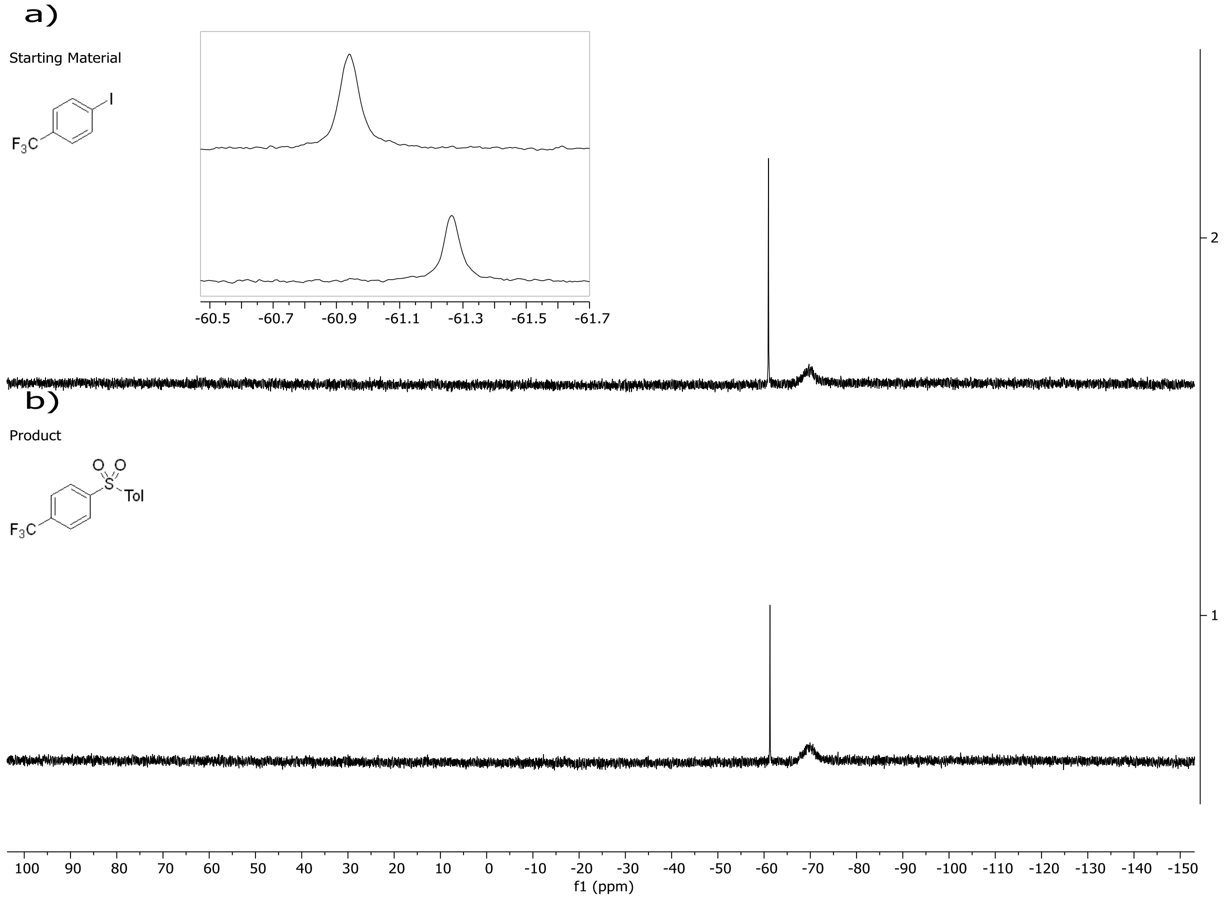

Figure S3: Benchtop ^{19}F-NMR spectra of (a) starting material, 4-iodobenzotrifluoride (b) product **1**. The broad peak at −70 ppm is the internal lock of the spectrometer.

To evaluate the accuracy and precision of the in-line analysis, 2 mL of reaction solution collected during steady state was measured by 375 Hz ^{19}F NMR (Varian 400 spectrometer) with hexafluorobenzene as an internal standard. The results show minor different between two analytic methods (Table S1), indicating the results collected by the in-line are trustable.

Table S1: Comparison on in-line benchtop and off-line high-field NMR

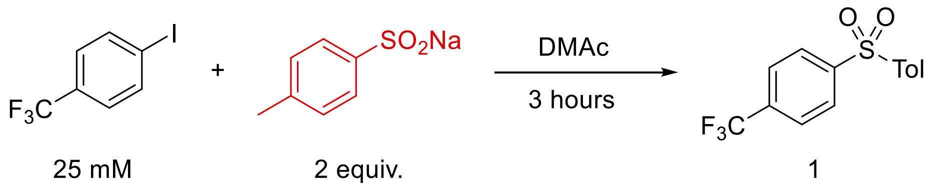

Entry	Analytic method	Conversion [%]	Yield [%]
1	In-line analysis	45	37
2	Off-line analysis	43	37

5. Reaction optimization

5.1. Flow setup (injection loop vs. continuous operation)

For the experiments performed with the injection loop, a 2.0 ml PTFE tubing loop was connected to the 6-way valve of the Vapourtec R-series reactor and the reaction mixture was loaded on the loop with a syringe and injected in the stream of solvent (DMAc) pumped by a syringe pump to the reactor. On the other hand, for the experiment under continuous-flow conditions, the reaction mixture was directly pumped from the syringe loaded on the syringe pump as described in the general reaction procedure.

5.2. Conversion vs time

The data for Figure 1 of the manuscript is presented in tabular format in Table S2.

Table S2: Comparison on coupling of 4-iodobenzotrifluoride and sodium *p*-toluenesulfinate.

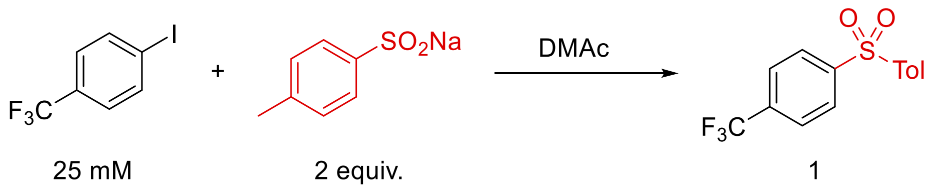

Entry[a]	Reaction time [h]	Flow rate [µL/min]	Conversion [%]	Yield [%]	Selectivity [%]
1	0.3	50	13	8	62
2	1	25	22	19	87
3	3	11	43	37	86
4	5	6	69	42	61
5	10	3	86	70	81
6	15	2	92	79	85

[a] Conversion and yield are calculated by (high-field, 376 MHz) ^{19}F NMR.

5.3. Optimization of temperature and light intensity

The effect of temperature and light intensity on the reaction were evaluated by one-variable-at-a-time method, i.e. all the other parameters were kept constant while changing either temperature or light intensity. High temperature darkened the material in the packed-bed column (Figure S4).

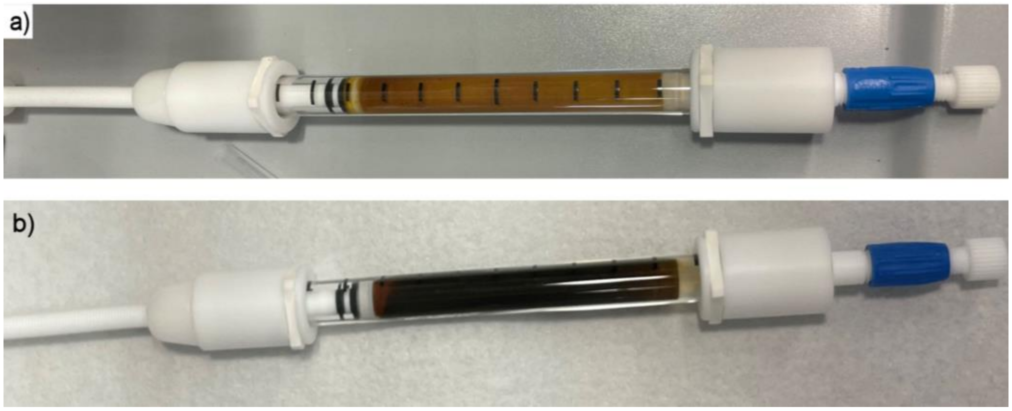

Figure S4: Photos of the packed column (a) before reaction (b) after irradiation at 70 °C for 12 hours.

5.4. Catalyst loading

The impact of the catalyst loading was evaluated by preparing packed bed with different poly-czbpy content, ranging between 3.3 and 0.8% w/w. A trend for higher selectivity with lower catalyst loading is observed, while the change in conversion was relatively minor and could partly be attributed to the difference in packed bed volumes. We selected the ratio of 1.6% for further experiment since it balances the amount of poly-czbpy needed and the selectivity while maintaining essentially the same activity of the 3.3% w/w loading.

Table S3 Optimization of catalyst weight ratio for coupling of 4-iodobenzotrifluoride and sodium *p*-toluenesulfinate.

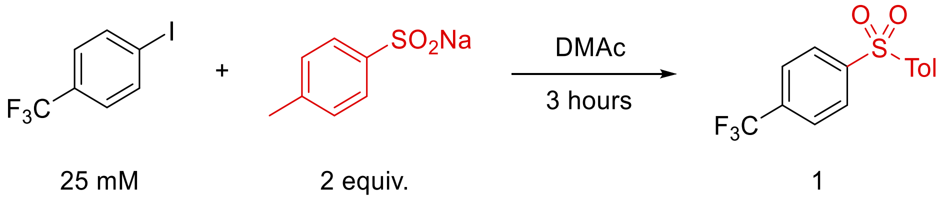

Entry[a]	poly-czbpy ratio [% w/w]	Conversion [%]	Yield [%]	Selectivity [%]
1	3.3	46	36	78
**2**	**1.6**	**45**	**37**	**82**
3	1.2	38	34	89
4	0.8	35	32	91

[a] Data was collect by 37.6 MHz ^{19}F NMR

6. Residence Time Distribution analysis

The residence time distribution was determined by pulsed input experiment injecting acetone as a tracer. The experiment was carried out by injecting 75 µL of acetone with the sample loop of a 6-way valve (Knauer V2.1S). In particular, acetone was injected in the stream generated by a syringe pump (Harvard Apparatus Elite 11) set on the same flowrate used for the majority of the experiments, i.e. 10 µL/min. The mean residence time and variance were monitored with the absorption at 305 nm with an HPLC PDA detector (Knauer PLATINblue, Figure S5) and calculated by custom Python script (Supporting Information File 2).

The distribution showed no difference Omnifit column with and without mixer. Addition of static mixers to the column did not improve the mixing efficiency. 5/32” i.d. tube had better mixing efficiency, corresponding to the performance of better conversion of the reaction.

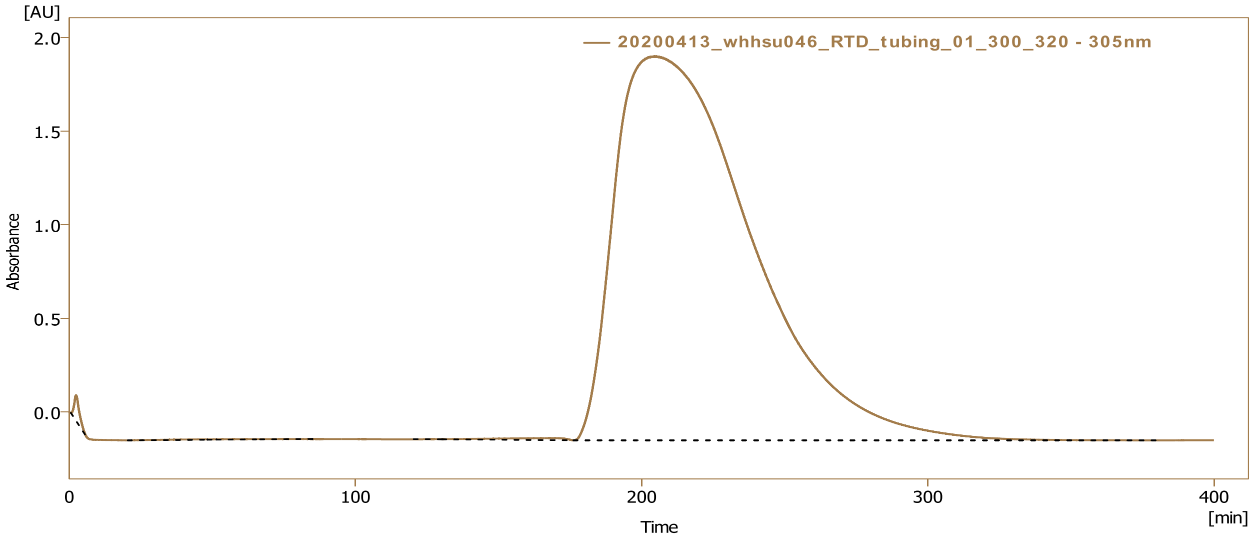

Figure S5: UV trace of the RTD experiment with pulse tracer in the 5/32” i.d. tubing reactor.

7. Nickel content

Overall 1973 mg of 1.6 wt % poly-czbpy mixture was used for the stability test (i.e., 33 mg of poly-czbpy). 7.54 mg of $NiCl_2$·glyme (measured by precision balance) dissolved in 8 mL DMAc and recirculated through the packed bed with an HPLC pump (Knauer P4.1S)

a flow rate of 0.5 mL/min for 3 hours (residence time ≅ 5 min). 30 mL of DMAc were used to wash the packed bed after the recirculation. The initially nickel amount after complexation was quantified by weight of initially loading amount of nickel species and the remaining amount of nickel in the washing solution after complexation. 7 days reaction solution was collected and then evaporated under reduced pressure. The residue was analysed by ICP-OES, resulting in 416.7 μg of nickel was leached (256 ppm over 1625 mg as an average of three samples, %CV 2.3) in the long-run experiment.

8. Compound characterization

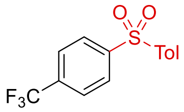

1

4-Tosylbenzotrifluoride (1)

Obtained from 4-iodobenzotrifluoride and sodium *p*-toluenesulfinate according to the general procedure with the 5/32” i.d. tube reactor (conditions as per Table 2 entry 3). Purification by flash chromatography (1:10 ethyl acetate to hexane) after evaporating the reaction solvent under reducing pressure afforded the title compound as a white solid. R_f value: 0.4 (20% ethyl acetate/hexane). NMR yield: 50%, isolated yield: 43% (6.4 mg over 2.0 mL of reaction mixture). ^{1}H-NMR (400 MHz, $CDCl_3$) δ 8.05 (d, J = 8.1 Hz, 2H), 7.84 (d, J = 8.3 Hz, 2H), 7.75 (d, J = 8.3 Hz, 2H), 7.33 (d, J = 8.1 Hz, 2H), 2.41 (s, 3H). ^{13}C-NMR (101 MHz, $CDCl_3$) δ 145.71, 145.08, 137.70, 134.77 (q, J = 33.1 Hz), 130.31, 128.17, 128.08, 126.51 (q, J = 3.7 Hz), 123.25 (q, J = 273.1 Hz), 21.75. ^{19}F-NMR (376 MHz, $CDCl_3$) δ -63.19. The compound characterization is in agreement with literature data[1].

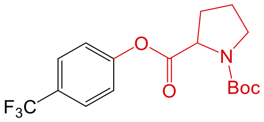

2

1-(*tert*-Butyl) 2-(4-(trifluoromethyl)phenyl)pyrrolidine-1,2-dicarboxylate (2)

Reaction solution prepared with 4-iodobenzotrifluoride (50 mM), *N*-(*tert*-butoxycarbonyl)-L-proline (*N*-Boc-proline, 75 mM, 1.5 equiv), and *N*-*tert*-butylisopropylamine (BIPA, 150 mM, 3 equiv) in dimethyl sulfoxide (DMSO). 2 mL of reaction mixture (i.e., approximately 31 mg starting material, 4-iodobenzotrifluoride) were collected from the reactor outlet after steady state conditions had been reached. Purification by flash chromatography (1:10 ethyl acetate/ hexane) after evaporating the reaction solvent and extraction by ammonium chloride$_{(aq)}$ and brine. The product was obtained as a colorless oil. R_f value: 0.24 (20% ethyl acetate/hexane). Rotameric mixture. ^{1}H NMR (400 MHz, $CDCl_3$) δ 7.67 (m, 2H), 7.28 – 7.24 (m, 2H, contains residual solvent signal of $CDCl_3$), 4.57– 4.44 (m, 1H), 3.67

– 3.47 (m, 2H), 2.46 – 2.37 (m, 1H), 2.22 – 2.15 (m, 1H), 2.10 – 1.96 (m, 2H), 1.48 (m, 9H). ^{13}C NMR (101 MHz, $CDCl_3$) rotameric mixture, resonances for minor rotamer are enclosed in parenthesis δ (171.27) 171.16, (154.51) 153.63, (153.31) 153.06, 128.17 (d, J = 32.6 Hz) (127.99 (d, J = 32.8 Hz)), 126.91 (q, J = 3.7 Hz) (126.74 (q, J = 3.8 Hz)), (122.03) 121.65, 80.39 (80.20), 59.17 (59.08), (46.66) 46.48, 31.05 (29.99), 29.72, 28.42, (24.59) 23.76. ^{19}F-NMR (376 MHz, $CDCl_3$) δ -62.22, -62.27. The compound characterization is in agreement with literature data[2]**.**

9. References

(1) Chen, L.; Liang, J.; Chen, Z.; Chen, J.; Yan, M.; Zhang, X. *Advanced Synthesis & Catalysis* **2019**, *361*, 956–960. doi:10.1002/adsc.201801390

(2) Pieber, B.; Malik, J. A.; Cavedon, C.; Gisbertz, S.; Savateev, A.; Cruz, D.; Heil, T.; Zhang, G.; Seeberger, P. H. *Angewandte Chemie International Edition* **2019**, *58*, 9575–9580. doi:10.1002/anie.201902785

10. NMR spectra

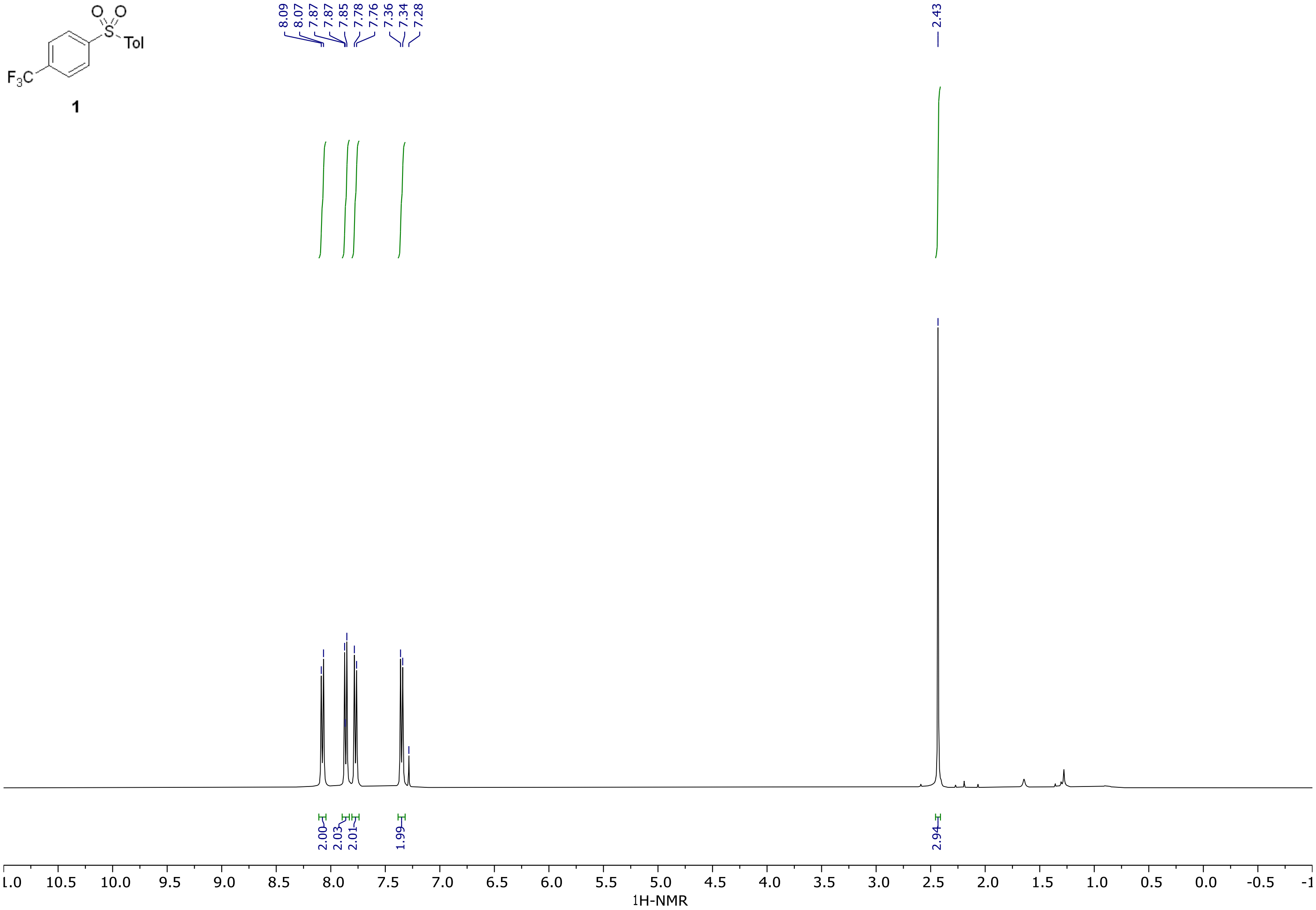

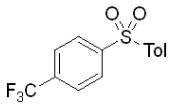
1

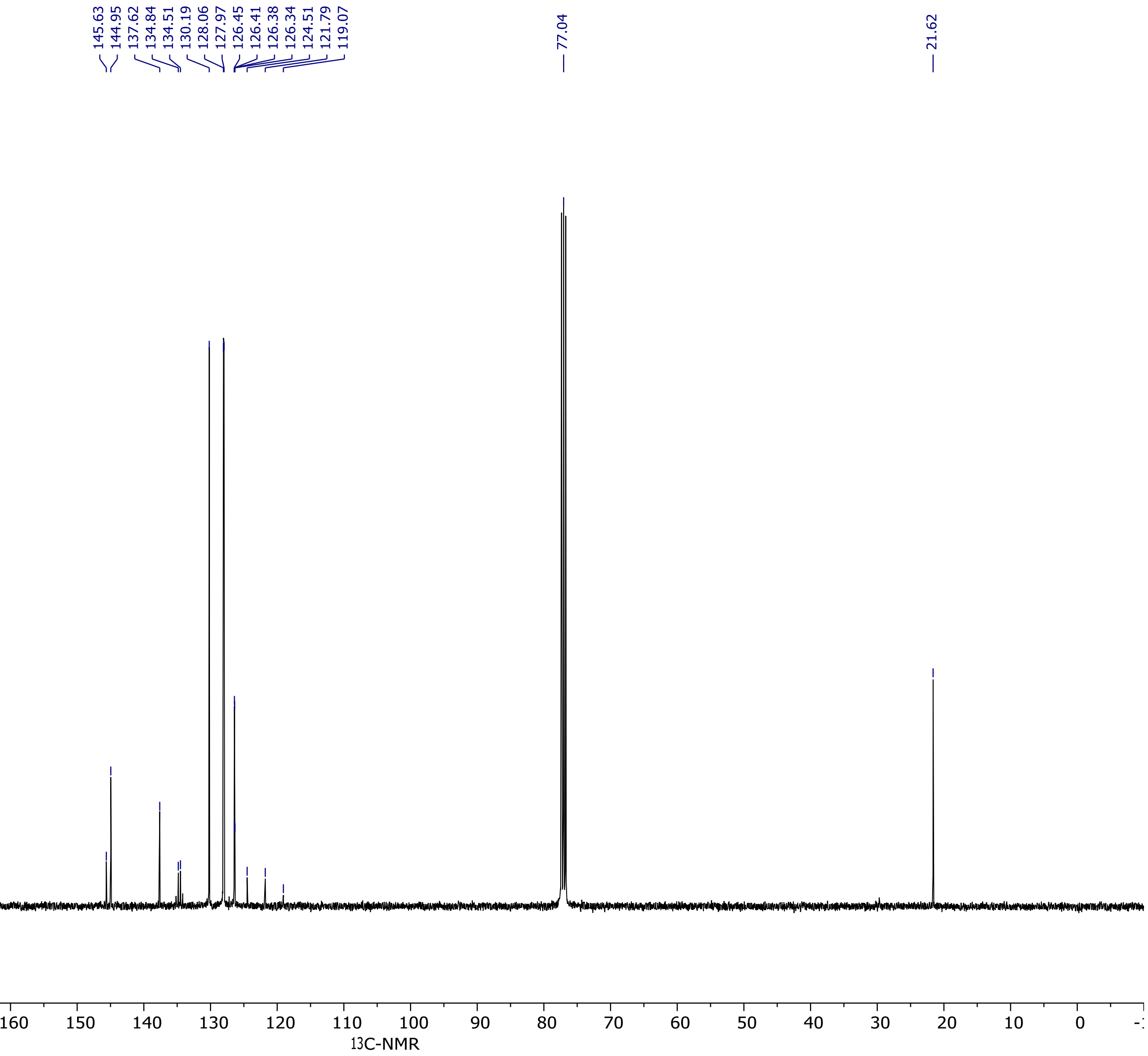

145.63
144.95
137.62
134.84
134.51
130.19
128.06
127.97
126.45
126.41
126.38
126.34
124.51
121.79
119.07
77.04
21.62
210 200 190 180 170 160 150 140 130 120 110 100 90 80 70 60 50 40 30 20 10 0
13C-NMR

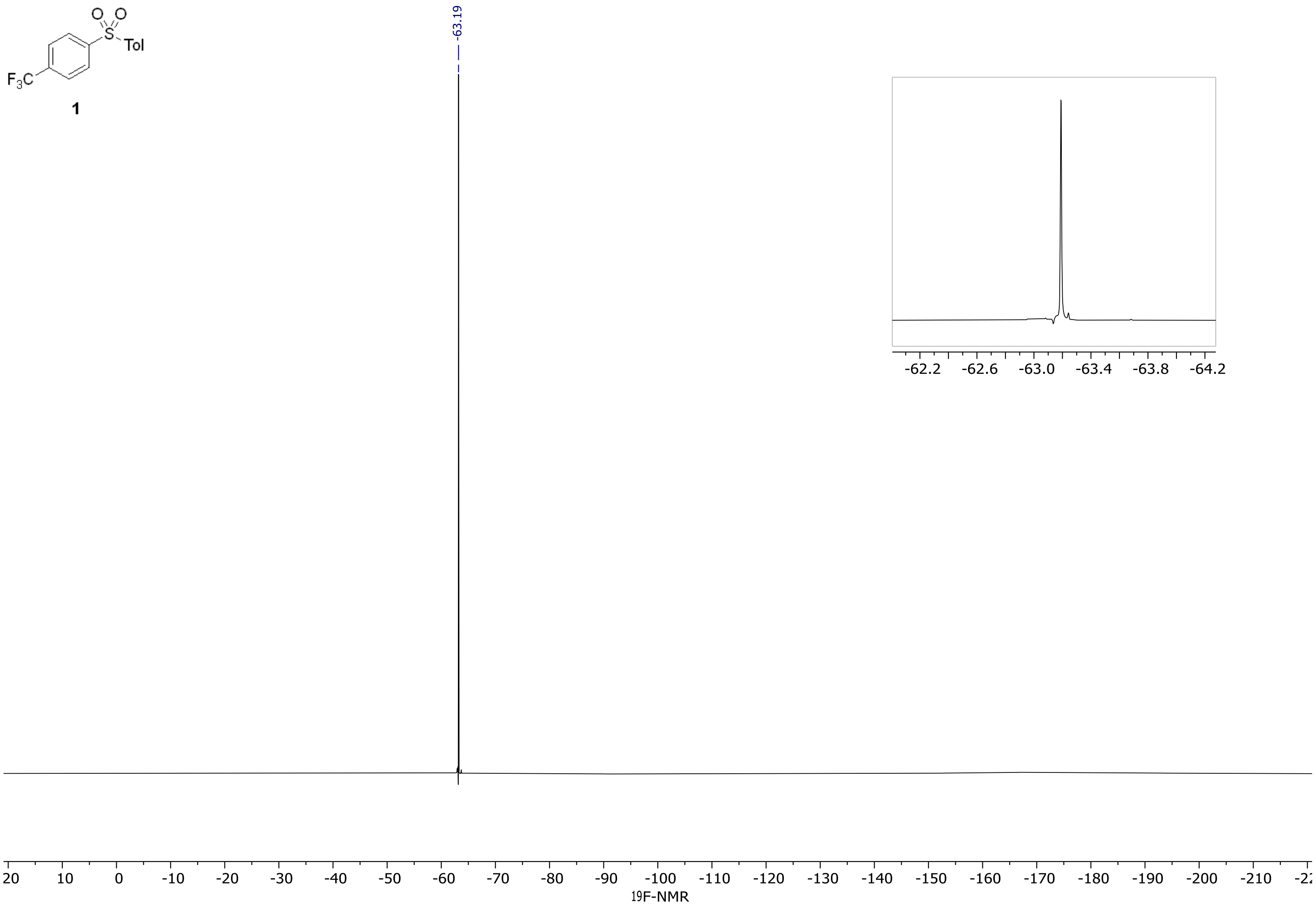

F3C
Tol
1
-63.19
-62.2
-62.6
-63.0
-63.4
-63.8
-64.2
20
10
0
-10
-20
-30
-40
-50
-60
-70
-80
-90
-100
-110
-120
-130
-140
-150
-160
-170
-180
-190
-200
-210
19F-NMR

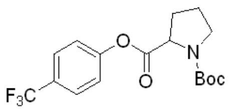

2

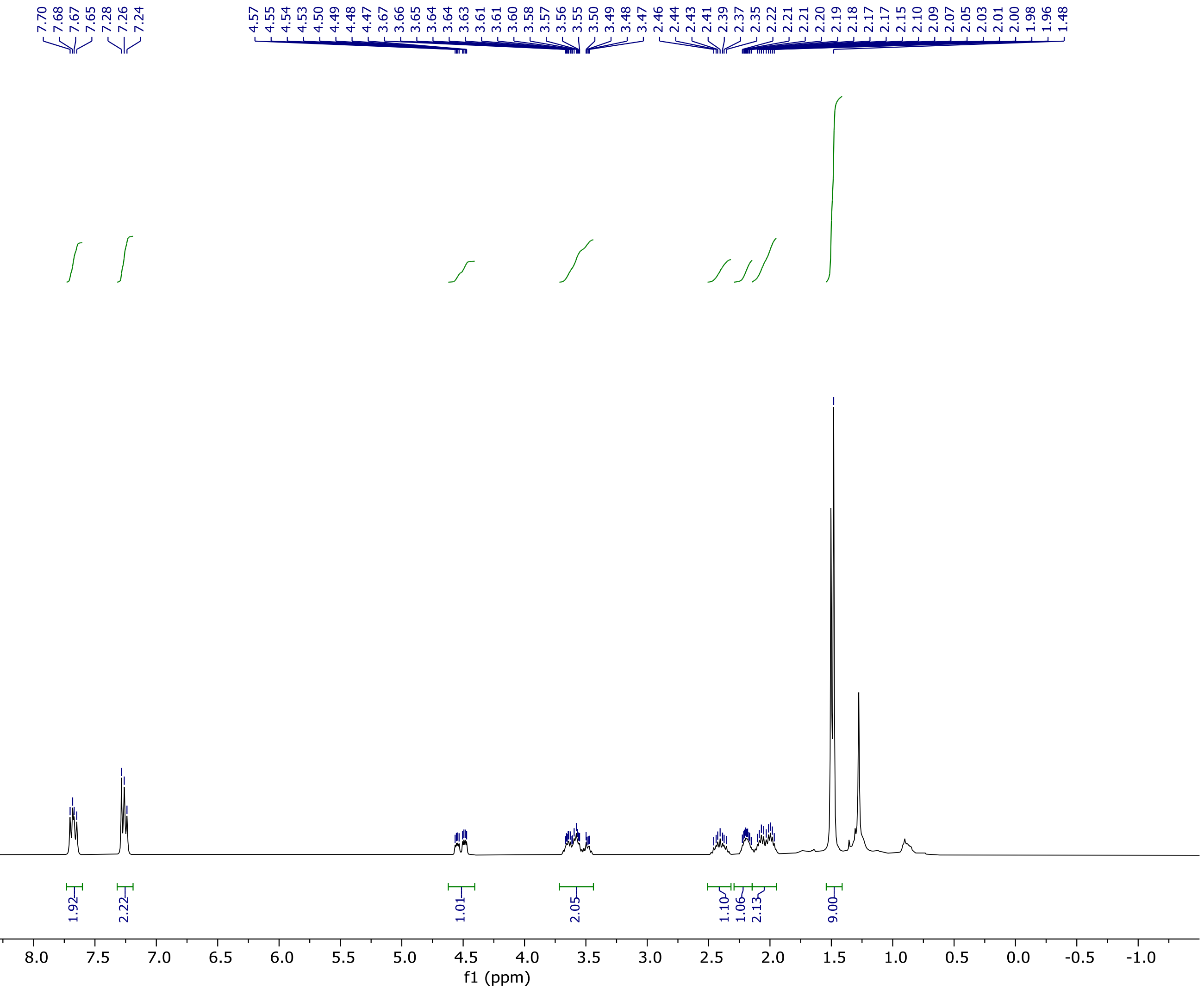

7.70
7.68
7.67
7.65
7.28
7.26
7.24
4.57
4.55
4.54
4.53
4.50
4.49
4.48
4.47
3.67
3.66
3.65
3.64
3.64
3.63
3.61
3.61
3.60
3.58
3.57
3.56
3.55
3.50
3.49
3.48
3.47
2.46
2.44
2.43
2.41
2.39
2.37
2.35
2.22
2.21
2.21
2.20
2.19
2.18
2.17
2.17
2.15
2.10
2.09
2.07
2.05
2.03
2.01
2.00
1.98
1.96
1.48
1.92
2.22
1.01
2.05
1.10
1.06
2.13
9.00
f1 (ppm)

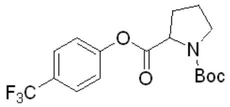

F3C
O
O
N
Boc

2

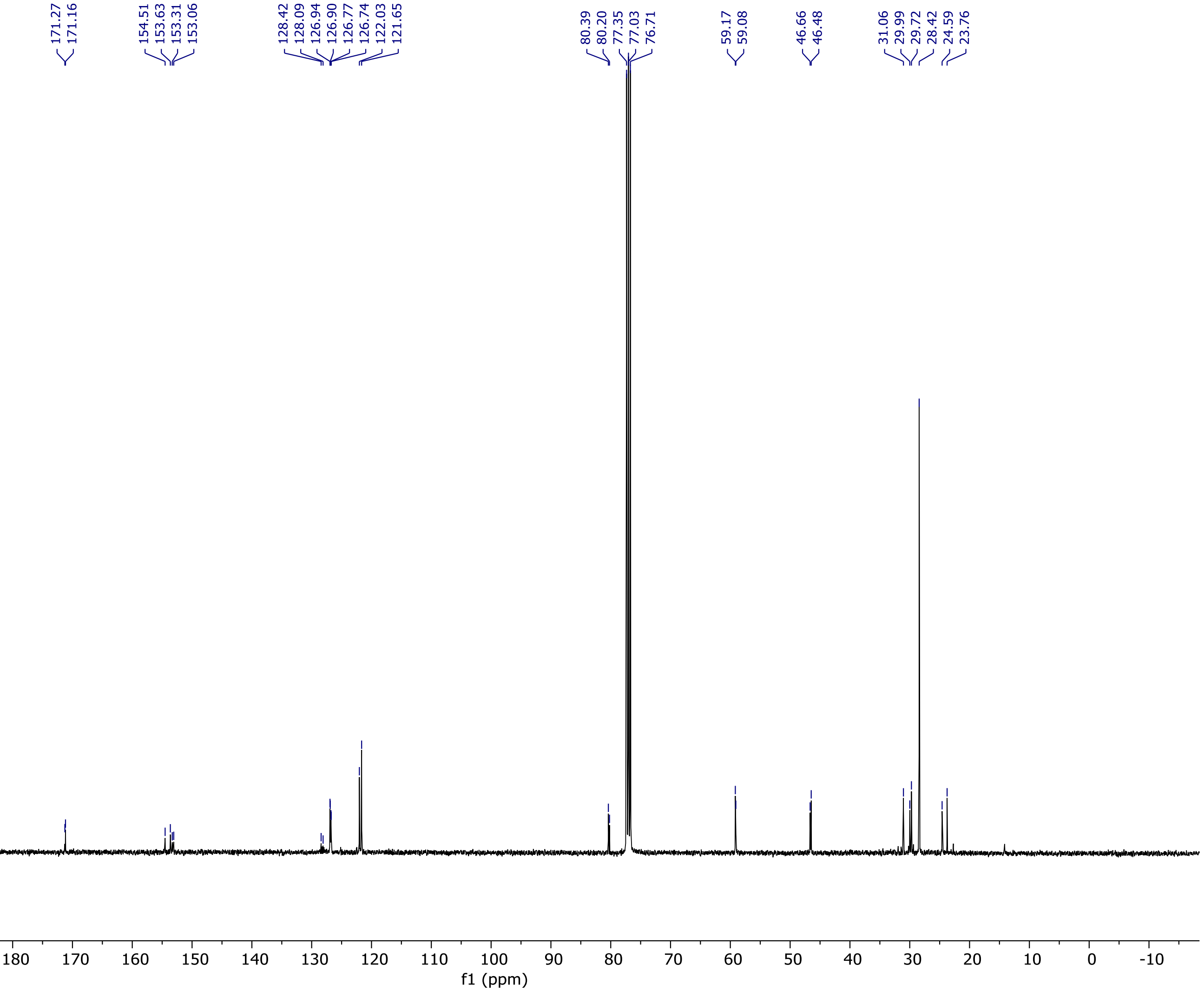

171.27
171.16
154.51
153.63
153.31
153.06
128.42
128.09
126.94
126.90
126.77
126.74
122.03
121.65
80.39
80.20
77.35
77.03
76.71
59.17
59.08
46.66
46.48
31.06
29.99
29.72
28.42
24.59
23.76
210 200 190 180 170 160 150 140 130 120 110 100 90 80 70 60 50 40 30 20 10 0 -10
f1 (ppm)

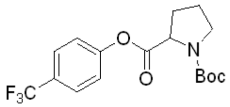

F3C
O
O
N
Boc

2

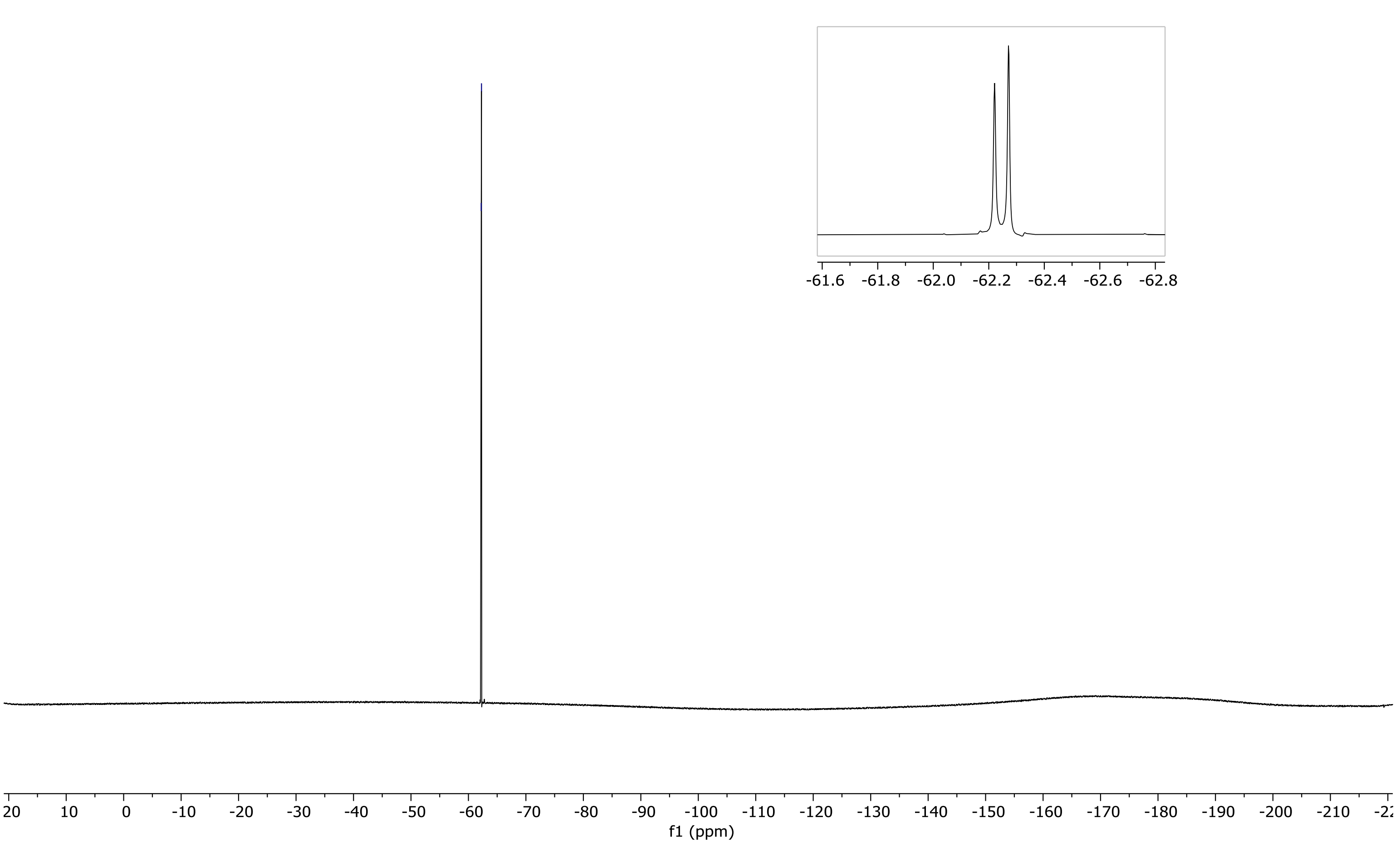

-62.22
-62.27
-61.6
-61.8
-62.0
-62.2
-62.4
-62.6
-62.8
20
10
0
-10
-20
-30
-40
-50
-60
-70
-80
-90
-100
-110
-120
-130
-140
-150
-160
-170
-180
-190
-200
-210
f1 (ppm)
