# Supplementary material for: Heterogeneous metallaphotoredox catalysis in a continuous-flow packed-bed reactor
Source: Beilstein J Org Chem. 2022 Aug 29;18:1123–30. doi: 10.3762/bjoc.18.115 (PMC9443413; doi:10.3762/bjoc.18.115)
Supplement: File 2 — Residence time distribution calculation notebook. [file Beilstein_J_Org_Chem-18-1123-s002.zip › SI2_RTD calculation/SI2_RTD calculation.pdf]

SI2_RTDcalculation

June 21, 2022

In [22]:
```
# Plots the residence time distribution from raw data
# Based on http://websites.umich.edu/~elements/course/lectures/thirteen/index.htm

import math
import pandas
from pathlib import Path
import numpy as np
%matplotlib inline
```

In [23]:
```
# Give the path to the csv-file with the tracer experiment
tracer_xp = Path(
    r"W:\BS-FlowChemistry\People\Wei-Hsin\RTD_tubing_305nm.txt"
)

# Read it into a Pandas Dataframe
experimental_data = pandas.read_csv(tracer_xp, header=16, sep='\t')
# (rename dataframe columns for clarity)
experimental_data.columns = ["Time [min]", "Absorbance [A.U.]"]
# Plot curve
experimental_data.plot(x=0, y=1, title="Experimental concentration curve")
```

Out[23]:
```
<matplotlib.axes._subplots.AxesSubplot at 0x2533dc8df28>
```

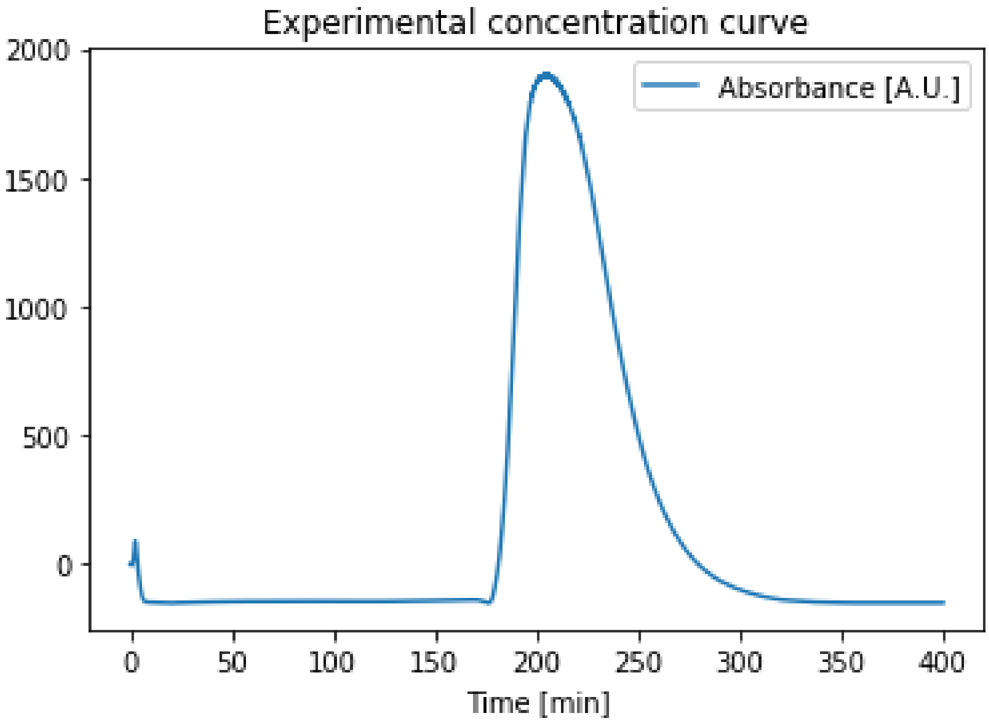


```
In [24]: # Baseline correction
         min_value = pandas.Series.sort_values(experimental_data["Absorbance [A.U.]"],
                                                ascending = True)[:200].mean()
         experimental_data['corrected_signal'] = \
             experimental_data["Absorbance [A.U.]"] - min_value

In [25]: # Nomolization: get the AUC of the experimental concentration curve:
         auc = np.trapz(
             experimental_data["corrected_signal"],
             x=experimental_data["Time [min]"]
         )

         # the exit age curve E(t) is obtained dividing the Y-axis by the AUC (total integral
         experimental_data['E'] = experimental_data["corrected_signal"] / auc

         # Plot curve
         experimental_data.plot(x=0, y=2, title="Exit age curve")

Out[25]: <matplotlib.axes._subplots.AxesSubplot at 0x2533dc85f60>
```

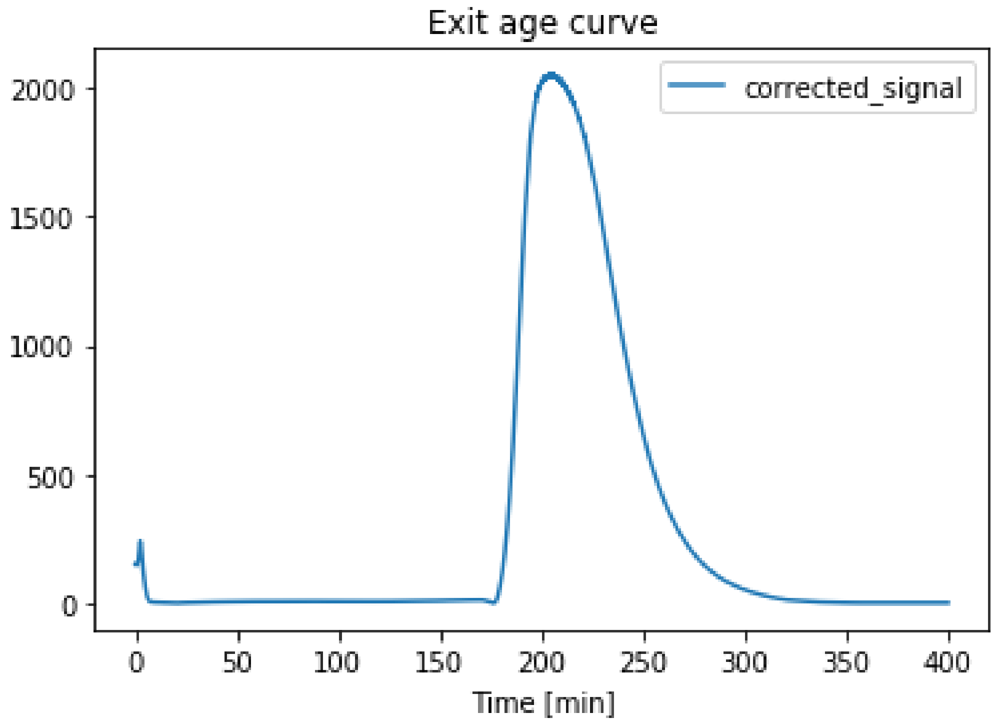


```
In [26]: mean_time = np.trapz(
             experimental_data['E'] * experimental_data["Time [min]"],
             x=experimental_data["Time [min]"])/ np.trapz(experimental_data['E'],
                                                     x=experimental_data["Time [min]"])
         variance = np.trapz(
             (experimental_data["Time [min]"] - mean_time) ** 2 * experimental_data['E'],
             x=experimental_data["Time [min]"])

         sigma = math.sqrt(variance)

         print(f'The mean residence time tau is: {mean_time:.3f} ś {sigma:.3f}.')
```

```
The mean residence time tau is: 217.882 ś 32.188.
```
