# Supplementary material for: Heterogeneous metallaphotoredox catalysis in a continuous-flow packed-bed reactor
Source: Beilstein J Org Chem. 2022 Aug 29;18:1123–30. doi: 10.3762/bjoc.18.115 (PMC9443413; doi:10.3762/bjoc.18.115)

NMRcontrol_autointegration

June 21, 2022

```
In [8]: import asyncio
        import numpy as np
        import pandas as pd
        import matplotlib.pyplot as plt
        import itertools as it
        from flowchem.components.devices.Magritek import Spinsolve, NMRSpectrum
        from flowchem import Knauer16PortValve
```

```
In [ ]: # Acquisition and integration settings

        # set the wait time between two NMR measurement
        NMR_DELAY = 60 * 2  # in sec
        counter = it.count()

        # set the integration limits (in ppm)
        peak_list = np.array(
            [['SM', -60.85, -61.1 ],
             ['Product', -61.1 , -61.35],
             ['Side-P', -60.5 , -60.7 ]]
        )
```

```
In [ ]: async def Analysis(observed_result):
            nmr = Spinsolve(host="BSMC-YMEF002121")

            while True:
                path = await nmr.run_protocol(
                    "1D FLUORINE+",
                    {
                        "Number": 128,
                        "AcquisitionTime": 3.2,
                        "RepetitionTime": 2,
                        "PulseAngle": 90,
                    },
                )
                observed_time = (NMR_DELAY / 60 + 4) * next(counter)

                peak_normalized_list = peak_aquire_process(path)
```

```
                observed_result = observed_result.append(
                    pd.DataFrame(
                        peak_normalized_list,
                        index=["SM", "product", "side-P"],
                        columns=[observed_time],
                    ).T
                )

                # Print results
                print(observed_result)

                # save to CSV
                observed_result.to_csv(
                    r"W:\BS-FlowChemistry\People\Wei-Hsin\Spinsolve\dataframe_0317_03.csv",
                    header=True,
                )

                plt.figure()
                observed_result.plot()
                plt.legend(loc="best")
                plt.savefig(
                    r"W:\BS-FlowChemistry\People\Wei-Hsin\Spinsolve\plot_0317_03.png"
                )

                await asyncio.sleep(NMR_DELAY)
```

In [ ]:
```
def peak_aquire_process(path):
    spectrum = NMRSpectrum(path)
    spectrum.process()

    peak_sum_list = []

    # loop over the integration limits
    for name, start, end in peak_list:
        min = spectrum.uc(start, "ppm")
        max = spectrum.uc(end, "ppm")
        if min > max:
            min, max = max, min
        # extract the peak
        peak = spectrum.processed_data[min : max + 1]
        peak_sum_list.append(peak.sum())

    # peak normalization
    y = sum(peak_sum_list)
    peak_normalized_list = [i / y for i in peak_sum_list]
    return peak_normalized_list
```

In [ ]:
```
async def main():
    observed_time = 0
```

```
        observed_result = pd.DataFrame(
            [1, 0, 0], index=["SM", "product", "side-P"], columns=[observed_time]
        ).T
        await Analysis(observed_result)


if __name__ == "__main__":
    asyncio.run(main())
```

0.1 Example results

A panda dataframe from an experiment is used to show examplary results.

```
In [7]: %matplotlib inline
        result_file = r"220405_in-line_whhsu044_rate10uLmin_50_02.csv"
        df = pd.read_csv(result_file)

        df.plot(x=0, xlabel= 'Time (min)')
```

```
Out[7]: <matplotlib.axes._subplots.AxesSubplot at 0x2d4f65e1c50>
```

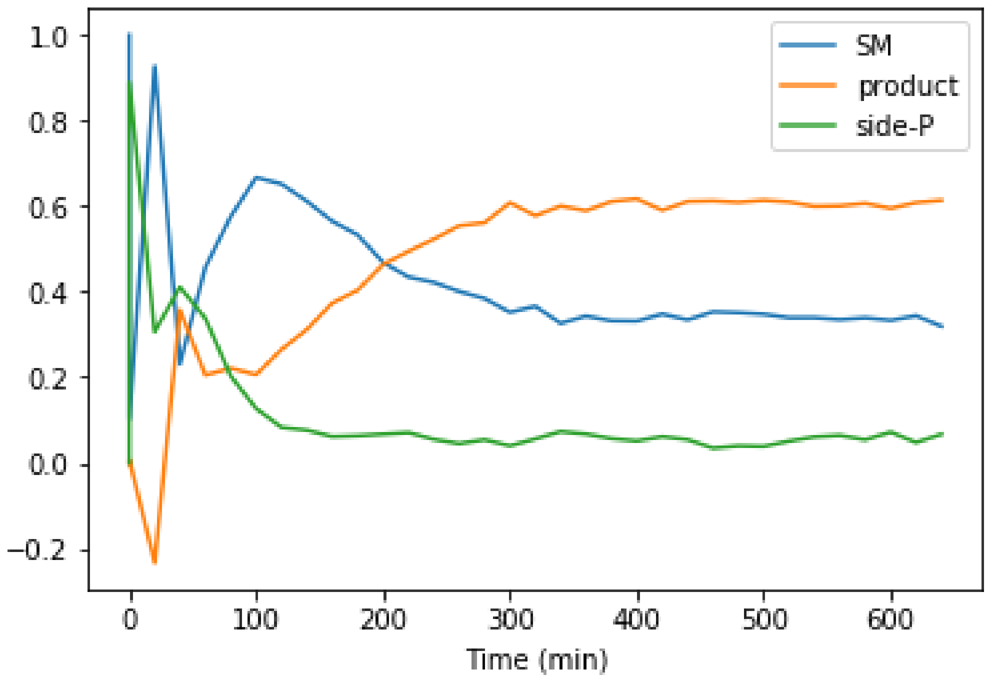

Supplement: File 3 — NMR control and auto-integration notebook. [file Beilstein_J_Org_Chem-18-1123-s003.zip › SI3_NMR control_autointegration/SI3_NMR control_autointegration.pdf]
